# The value of intraovarian autologous platelet rich plasma in women with poor ovarian reserve or ovarian insufficiency: a systematic review and meta-analysis

**DOI:** 10.1186/s12884-024-06251-2

**Published:** 2024-01-27

**Authors:** Ahmed M. Maged, Reham A. Mohsen, Noha Salah, Wael S. Ragab

**Affiliations:** 1 Department of Obstetrics and Gynecology, Kasr Al-Ainy Hospital, Cairo University, Cairo, Egypt; 2 Department of Obstetrics and Gynecology, Faculty of Medicine, Fayoum University, Fayoum, Egypt

**Keywords:** Platelet-rich plasma, PRP, Autologous platelet-rich plasma, Poor ovarian reserve, Ovarian insufficiency

## Abstract

**Objectives:**

To assess the value of intraovarian PRP in women with low ovarian reserve.

**Search strategy:**

Screening of databases from inception to January 2023 using the keywords related to “Platelet-rich plasma” AND “poor ovarian reserve” OR “ovarian failure”.

**Selection criteria:**

Fourteen studies (1632 participants) were included, 10 included women with POR, 1 included women with POI and 3 included both POR and POI women.

**Data collection and analysis:**

Extracted data included study settings, design, sample size, population characteristics, volume, timing and preparation of PRP administration, and outcome parameters.

**Main results:**

AMH level was evaluated in 11 studies (2099 women). The mean difference (MD) was 0.09 with 95% CI of – 0.06, 0.24 (*P* = 0.25). Antral follicular count level was assessed in 6 studies (1399 women). The MD was 1.73 with 95% CI of 0.81, 2.66 (*P* < 0.001). The number of oocytes retrieved was evaluated in 7 studies (1413 women). The MD was 1.21 with 95% CI of 0.48, 1.94 (*P* = 0.001).

**Conclusion:**

This systematic review found a significant improvement of AFC, the number of retrieved oocytes, the number of cleavage embryos and the cancellation rate in women with POR.

**Trial registration:**

Registration number CRD42022365682.

**Supplementary Information:**

The online version contains supplementary material available at 10.1186/s12884-024-06251-2.

## Synopsis

Intraovarian PRP injection improved AFC, the number of retrieved oocytes, the number of cleavage embryos and the cancellation rate in women with POR.

## Introduction

The ovary is considered as the biological clock that control the aging process in the female [1].

Ovarian aging is defined as gradual decrease in oocyte quality and quantity and eventful ovarian function exhaustion [2].

Two types of ovarian aging are known. Physiological aging is the natural deterioration of ovarian function with age that end in menopause, while pathological aging is the premature diminishment of ovarian function as a result of certain pathogenic factors [3].

Pathological ovarian aging includes premature ovarian insufficiency (POI), diminished ovarian reserve (DOR) and poor ovarian response (POR) for controlled ovarian hyperstimulation (COH) [4].

POR is not uncommonly encountered during COH. Its prevalence is between 5 and 35% of women with subfertility. It is defined as failure of the ovary to respond adequately to standard ovarian induction protocols and production of adequate ova. It is one of the rate limiting steps in success of IVF that is characterized by low or even failure of oocyte retrieval, higher rates of cycle cancelation and the lower probability of pregnancy [5].

Many interventions have been suggested to improve the outcome of COH in POR. These include pretreatment with aromatase inhibitors, human chorionic gonadotropin or androgens [6]; adjuvant treatment with estrogen agonists, luteinizing hormones [7]; starting with the maximum dose of gonadotropin [8]; or the use of alternative protocols as microdose flare up [9], short flare up, agonist stop [10], antagonist (standard or delayed start) [11] or luteal phase support using follicle stimulating hormone [12].

Currently, there is no definitive treatment to reestablish normal ovarian function in women with POI [13].

But there are treatments for associated symptoms, in addition to treatments for reduction of associated risks. These include hormonal therapy, calcium and vitamin D supplementation, regular physical activity, keeping healthy body weight and emotional support [14].

Platelet-rich-plasma (PRP) is prepared from fresh whole blood through its centrifugation. The resultant precipitate is free from both red and white blood cells and rich in cytokines and growth factors as VEGF, TGFβ and PDGF that are released from α-granules of activated platelets [15].

Due to its high regenerative and anti-inflammatory properties, PRP is used in numerous medical fields, including orthopedics and ophthalmology [16].

PRP was first used to improve refractory thin endometrium in IVF [17].

It is currently studied in women with implantation failure, intrauterine synechia and POI. However, the results of its use showed contradictory findings [18].

PRP is a novel technique used in gynecology. The results of its use for improving and restoring ovarian function are conflicting among different studies. There is no sufficient data to support or decline its use. This raises the need for a properly conducted meta-analysis to guide its use in women with inadequate ovarian response.

This systematic review and meta-analysis aimed to assess the effects of intraovarian PRP injection in women with POI and poor ovarian response.

## Material and Methods

The study protocol was prepared based on the Preferred Reporting Items for Systematic reviews and Meta-Analyses (PRISMA) guidelines for meta-analysis. The protocol was prospectively registered at PROSPERO with CRD42022365682 number.

### Eligibility criteria, information sources, search strategy

Two authors (AM, WSR) independently searched Medline, Embase, Web of Science, Scopus, the Cochrane Central Register of Controlled Trials electronic databases from inception to January 2023 using the keywords “Platelet-rich plasma” OR “PRP” OR “Autologous platelet-rich plasma”) AND “premature ovarian failure” OR “decreased ovarian reserve” OR “premature menopause” OR “premature ovarian insufficiency” and their MeSH terms (Supplementary Table S1). Direct contact with authors via email was done to provide any clarifications or additional data.

### Study selection

All published and unpublished studies without language limitations (whether published in English or other languages) that involved intraovarian PRP injection in women with inadequate ovarian response or ovarian insufficiency were searched for. This systematic review included all prospective and retrospective studies, whether quasiexperimental, case control or comparative pilot ones, that involved the intraovarian PRP injection in women with POI and / or POR. Subgroup analysis for quasi-experimental, retrospective and case control studies were done. Both transvaginal and laparoscopic injection routes were also included. Non-human,invitro (cell culture) studies, case reports and studies with non-clearly reported outcomes or non-clear methodology (and cannot be clarified by author correspondence) were excluded from the analysis.

### Data extraction

Two authors (AM and AO) independently assessed the titles and abstracts of all search results, then assessed the full articles of the related trials. Any disagreement between the 2 authors for inclusion or data extraction was discussed with other coauthors. Extracted data included study settings, design, participants’ characteristics and number, PRP preparation method, intervention time and technique, outcome parameters, trial registration and funding details. Contacting the authors to clarify any unclear data via email was done.

Outcome parameters included serum AMH, basal FSH, basal E2, antral follicular count, spontaneous pregnancy rate, number of oocytes retrieved, number of cleavage and good quality embryos, fertilization, cancelation, clinical pregnancy, chemical pregnancy and live birth rates.

### Assessment of risk of bias

The Newcastle–Ottawa scale (NOS) [19] quality assessment of Non-randomized studies was done. The NOS star system uses 3 main assessments: the selection of the exposed and non-exposed groups; the comparability of the groups (before and after assessment or cases and control); and the ascertainment of both exposure and outcome (proper follow up). Absent and unclear data were requested through authors contact.

The GRADE system was used to assess the quality of evidence [19]. GRADE included risk of bias in the included studies, inconsistency, indirectness, imprecision, and publication bias. Serious concerns in each item decrease the evidence by 1 level while very serious ones decrease the evidence by 2 levels.

The levels were classified as high, moderate, low or very low according to the presence of strong, moderate, low or very low evidence that the true effect is close to the effect estimate, respectively.

### Data synthesis

The mean difference with the corresponding 95% CI was calculated for continuous data. No meta-analysis was done for dichotomous data as a result of marked heterogeneity of the outcome parameters. The effect size was obtained using the random effect model through the Mantel-Hansel method.

The *I*^2^ statistic and Cochran’s Q test were used to assess the heterogeneity of the included studies. A *P*-value of < 0.05 in the Q-test or I^2^ > 40% is considered as significant. The Review Manager (RevMan) version 5.4.1 (The Nordic Cochrane Centre, Cochrane Collaboration, 2020, Copenhagen, Denmark) was used for all statistical analysis.

## Results

### Study selection

**Fig. 1:**
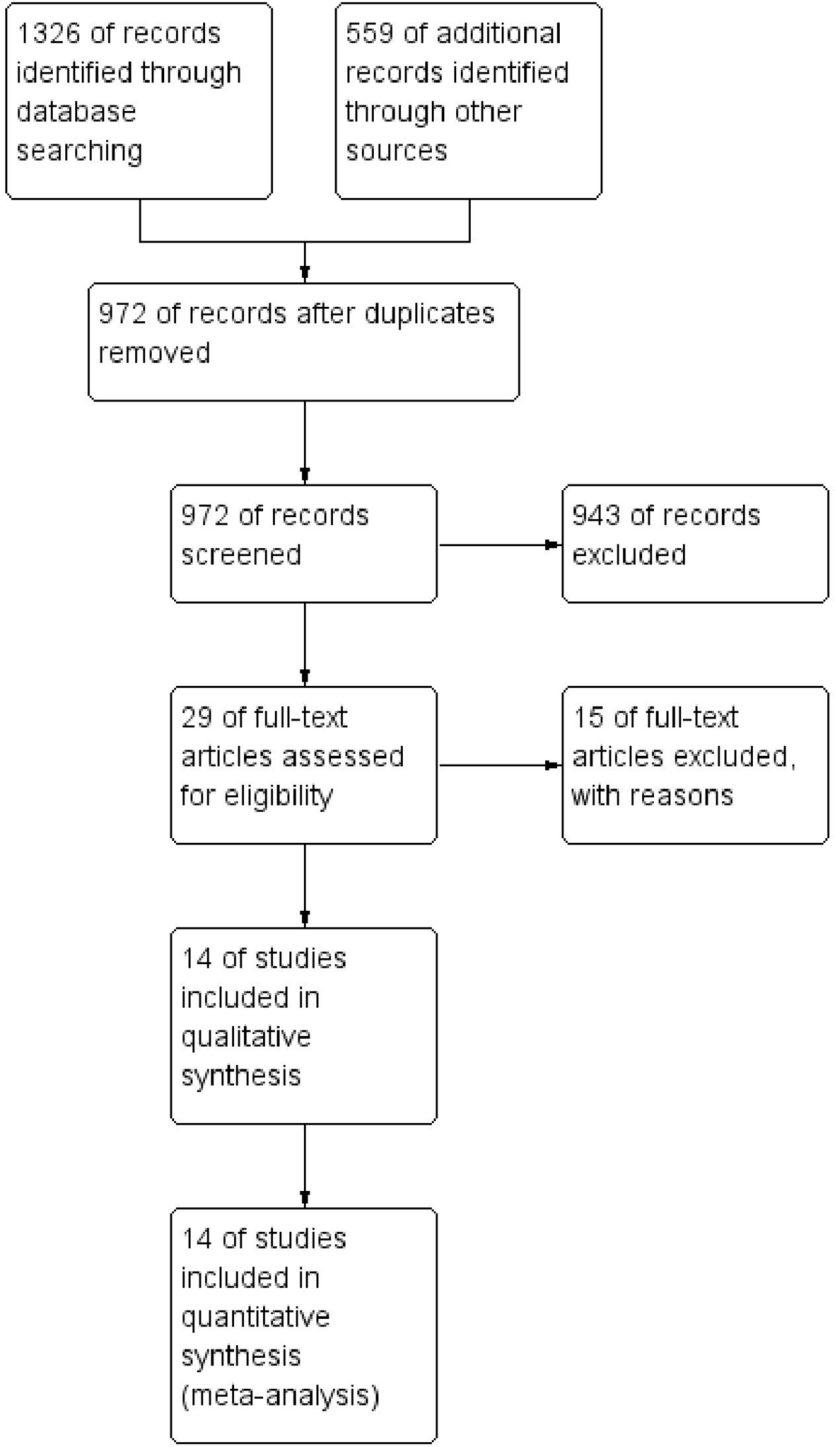
PRISMA flow chart

Our search yielded 1885 studies through databases (505 from PubMed, 113 from Embase, 624 from Scopus, 84 from Web of Science, and 559 from clinical trials), 972 of them were screened after removal of duplicates, 29 screened for full text, 14 studies were included in quantitative and qualitative synthesis (Fig. 1).

### Study characteristics

Table 1 summarized the main characteristics of the included studies.

**Table 1:** Characteristics of the included studies

Study	settings	Design	Size	Participants	PRP preparation	Injection	control	outcome	Registration	Funding
Aflatoonian 2021 [20]	Single center Iran	Quasi-experimental	26 17 POR 9 POI	POI (ESHRE: onset < 40 years, oligo-/amenorrhea ≥ 4 months, and FSH > 25 IU/l. POR (Bologna criteria: age > 40 years, history of POR ≤ 3 oocytes in previous stimulation, and low ovarian reserve tests (AMH < 1.1 ng/ml) or AFC < 5) Exclusion criteria BMI < 18 or > 30 kg/m2 above 30 or less than 18, autoimmune diseases, thrombophilia, sex chromosome abnormality, STDs, tubal factor infertility, endocrine disorders, endometriosis, previous major lower abdominal surgery and pelvic adhesions, renal failure, malignancy, abnormal semen Iatrogenic POI hormonal therapy within 1 month before or after PRP	Protocol: Rooyagen, Tehran, Iran manufacturer’s instruction Volume: 20 ml of peripheral venous Blood and 3 ml acid citrate A anticoagulant solution Centrifugation: 1600g for 10 Min → 3 layers bottom RBCs, a buffy coat layer, and supernatant cellular Plasma. The plasma layer and buffy coat were transferred to another tube and centrifuged at 3500g for 5 min to achieve 3 ml PRP Platelets concentration: 3—5 times higher than basal blood. Storage: at 4°C for1 h. Activation with calcium gluconate in a 1:9 ratio	Timing: random in amenorrheic POI and Day 10 of cycle in oligomenorrheic POI and POR Technique: Transvaginal ultrasound guided multifocal intramedullary infusion of 1.5 ml using a 17-gauge single lumen needle into each ovary under minimal sedation. Most women received a 2nd injection of 3 mL PRP 3 months after the 1st injection (5 POR women received 1 injection only as spontaneous conception occurred after the 1st injection Follow up duration was 1 year following 1st PRP injection	Before and after assessment	CPR ChPR Miscarriage Ovarian reserve markers	RCT20180818040828N2	Yazd Reproductive Sciences Institute, Shahid Sadoughi University of Medical Sciences, Yazd, Iran, under Grant Agreement No. 68876
Barad 2022 [21]	Single center USA	Quasi-experimental	80 extremely low functional ovarian reserve 54 regular menstruating and 26 oligomenorrhea	Age 44 -54 years, Previous poor response in previous IVF cycles (oocytes ≤ 3), FSH > 12 mIU/ml and/or AMH < 1.2 ng/ml. Exclusion criteria: a history of active autoimmune disease, ongoing anticoagulant therapy, or evidence of infection, blood diseases, thrombocytopenia or cancer	Protocol: Regen Lab PRP Kit (RegenLab America Inc., Montreal, Canada Volume: 10 ml whole blood drawn into the Regen Lab PRP vacutainer with gel separator and citrate Centrifugation: Twice. First for 10min at 3800 relative centrifugal force (RCF) and again for 5min at 1500 RCF. This results in platelets pellet on top of the gel and 4—5ml plasma above the gel. The upper plasma removed, and the tube was inverted 25 times to resuspend the platelets in the remaining plasma	Timing: random in amenorrheic POI and Day 3–5 of cycle in others Technique: Sub-cortical injections of 0.1ml of the PRP were repeated 7 to 12 times per ovary until 1.5 ml had been administered to each ovary using 20-gauge needle under ultrasound guidance FSH, E2 and follicular growth were monitored every 3 days for 2 weeks then weekly for another 2 weeks then monthly. COH for IVF was started 1 month after the PRP injection	Before and after assessment	Ovarian reserve markers CPR LBR	NCT04275700	intramural funds from The Center for Human Reproduction and the not-for-profit research Foundation for Reproductive Medicine
Cakiroglu 2020 [22]	Single center Turkey	Quasi-experimental	311 POI POI (ESHRE criteria: oligo/amenorrhea for ≥ 4 months, FSH > 25 IU/l on two occasions 4 weeks apart and onset before 40 years of age	Inclusion criteria Age: 24—40 years, Infertility for > 1 year, and having at least one ovary Exclusion criteria were history of malignancy, genetic ovarian insufficiency, prior major lower abdominal surgery with pelvic adhesions, anticoagulant use for which plasma infusion is contraindicated, and current or previous IgA deficiency	Protocol: T-lab autologous platelet-rich plasma kit (T-Biotechnology Laboratory, Bursa, Turkey) Volume: 20 mL blood sample Centrifugation: at 830 g for 8 min. A 16 G needle connected to a 5 ml syringe was rotated into the buffy coat layer To collect 2–4 cc then a second tube was processed similarly. 4—8 cc PRP was collected and transferred to the resuspension tube and shaken gently for 30 – 60 s	Time: random in amenorrheic POI and within 10 days 1–10 of cycle end in others Technique: Within 2 h of preparation, PRP injection was performed transvaginally under ultrasound guidance and under sedation anesthesia into at least one ovary into the subcortical and stromal areas using a 35 cm 17 G single lumen needle Expectant management for 6 weeks to allow spontaneous pregnancy or menses	Before and after assessment	CPR LBR Ovarian reserve markers	No	None
Cakiroglu 2022 [23]	Single center Turkey	Quasi-experimental	510 POR using POSEIDON criteria	Inclusion criteria: Age 30 – 45 years, a history of infertility for at least 1 year, and at least one ovary. Exclusion criteria: history of malignancy, prior major lower abdominal surgery resulting in pelvic adhesions, anticoagulant use for which plasma infusion is contraindicated, and current or previous IgA deficiency	Protocol: T-lab autologous platelet-rich plasma kit (T-Biotechnology Laboratory, Bursa, Turkey) Volume: 20 mL blood sample Centrifugation: at 830 g for 8 min. A 16 G needle connected to a 5 ml syringe was rotated into the buffy coat layer To collect 2–4 cc then a second tube was processed similarly. 4—8 cc PRP was collected and transferred to the resuspension tube and shaken gently for 30 – 60 s	Time: within 10 days 1–10 of cycle end in others Technique: Within 2 h of preparation, PRP injection was performed transvaginally under ultrasound guidance and under sedation anesthesia into at least one ovary into the subcortical and stromal areas using a 35 cm 17 G single lumen needle Expectant management for 6 weeks to allow spontaneous pregnancy or menses	Before and after assessment	CPR LBR Ovarian reserve markers	NCT04237909	None
Farimani 2021 [24]	Single center Iran	Retrospective	96 POR using POSEIDON criteria	Inclusion criteria: Any POR attending a single laboratory with the highest number of cases Exclusion criteria: Lack of follow-ups and incomplete laboratory results Diseases/disorders affecting the chance of fertility	Protocol: Shanghai protocol	Right after the first follicular puncture, the intra-ovarian PRP injection (2 ml) was performed under ultrasound guide followed by the second puncture for the second stimulation AMH, LH, E2 and FSH were measured after two menses	Before and after assessment	Ovarian reserve markers Oocyte retrieved CPR	No	None
Melo 2020 [25]	Single center Venezuela	Prospective controlled, non-randomized comparative study	83 low ovarian reserve 46 received PRP and 37 control	Inclusion criteria: Age ≥ 38 years Day 3 FSH > 12 mIU/mL AMH < 0.8 ng/m normal uterine cavity Exclusion criteria: Previous PID clinical/biochemical hyperandrogenism or polycystic ovaries tubal factor infertility, endometriosis, known platelet or thromboxane synthesis disorder known severe male factor	Volume: 5 blood collection tubes containing sodium citrate 3.8% were filled with 4.5 mL of blood each and centrifuged at 270 g for 10 min, then 100 μL of the platelet-rich supernatant were transferred from each of 4 of the original blood tubes and mixed with 0.1 mL of 10% calcium chloride The blood in the remaining fifth tube was not mixed with calcium chloride to allow for quantification of the total number of platelets	Timing: between days 7 and 9 of the menstrual cycle Technique: 200 μL of PRP were injected into the cortex of each ovary using a single lumen aspiration needle under transvaginal ultrasound guidance and sedation. Each ovary was punctured once only, with the single lumen needle being inserted into the ovarian cortex superficially, and a total of 200-μL PRP injected into the subcortical area of the ovary Follow up of all women for 12 months was done	37 no intervention	Ovarian reserve markers CPR LBR	No	None
Navali 2022 [26]	2 centers Iran	Quasi-experimental	35 POR criteria ( AMH < 1.1 ng/mL, AFC < 5–7, a history of cycle cancellation due to < 3 oocytes retreived	Inclusion criteria: infertile women Age 30—42 years with at least one ovary and, and willing cooperate. Exclusion criteria: FSH > 25, current or previous IgA deficiency, genital or non-genital cancers, anticoagulants treatment, chromosomal ovarian failure, prior pelvic surgery resulting in pelvic adhesions, anemia, (hemoglobin < 10 g/dl), thrombocytopenia, (platelet count < 10 5/μ)l and did not receive the PRP injection	Protocol: Royagen kit (Co. SN: 312,569, Arya Mabna Tashkis, Iran) Volume: 20 mL blood sample Centrifugation: at 830 g for 8 min. A 16 G needle connected to a 5 ml syringe was rotated into the buffy coat layer To collect 2–4 cc then a second tube was processed similarly. 4—8 cc PRP was collected and transferred to the resuspension tube and shaken gently for 30 – 60 s	Antibiotic administration before oocyte pickup and 1 h before PRP injection Technique: After oocyte pick up about 2 cc of PRP injected into the cortex of both ovaries using a 35 cm 17 G single lumen needle with Doppler monitoring to prevent large vessel injury. After 2 months or 3 menses, patients received a new ovarian stimulation cycle with the same way and dose	Before and after assessment	Ovarian reserve markers M II oocytes	No	Women’s Reproductive Health Research Centre, Tabriz University of Medical Sciences (grant number: 65746)
Pacu 2021 [27]	2 centers Romania	Retrospective	20 POR POSEIDON criteria	Inclusion criteria: Age 31—44 years Exclusion criteria: Male infertility, endocrine dysfunction, autoimmune diseases, thrombophilia, malignancies, infectious diseases, and a family history of neoplastic diseases	Protocol: EasyPRP kit; Neotec Biotechnology Ltd Volume: 60–80 venous blood Platelet count: 250,000–850,000 platelets/μl	Timing: between cycle day 3 and 5 Technique: 2–4 ml PRP at the level of the ovarian parenchyma, the approach of the ovary being at a distance from the vascular pedicle to avoid hemorrhagic accidents under general anaesthesia under ultrasonographic guidance (2 during laparoscpy) Follow up for 6 months was done	Before and after assessment	Ovarian reserve markers Cycle performance indicators	No	None
Petryk 2020 [28]	Single center Ukraine	Quasi-experimental	38 low ovarian reserve	Inclusion criteria: Age: 31–45 years Infertility with 2 or more failed oocyte recruitment during IVF cycles Have at least one normal ovary ≥ 1 ml volume Negative pregnancy test Exclusion criteria: significant chronic condition, cancer, or mental illness Ovarian or uterine lesions	Volume Two tubes. Each contains 8.5 ml venous blood + 1.5 trisodium citrate with citric acid and dextrose	Centrifugation: at a G-force of 800 for 3 min results in platelet-poor plasma which is then withdrawn into Falcon 15-ml conical centrifuge tubes. Recentrifugation for 15 min at room temperature at a G-force of 1400, the precipitate of platelets was obtained, and then 75% of the upper volume of PPP was withdrawn again. The platelet precipitate was resuspended in the remaining PPP resulting in 2 ml solution 0.7 ml of PRP was injected into each ovary with a concentration of 1,000,000 platelets per microliter (μl) using 25 G needle, 20 cm in length guided by ultrasound (In difficult cases, a laparoscopic-assisted approach was used)	Before and after assessment Follow up for 12 months was done		No	None
Sfakianoudis 2020 [29]	Single center Greece	Quasi-experimental	120 women 30 POR (Bologna Criteria) 30 POI (Age < 40 years, Amenorrhea for ≥ 4 months, and FSH > 25 IU/L) 30 perimenopase (Age < 40 years and Menstrual cycle irregularities) 30 menopausal (Age 45–55 years, Amenorrhea for ≥ 12 months, and FSH > 30 IU/L)	Inclusion criteria: BMI 18.5 – 30 kg/m2 Exclusion criteria: autoimmune disorders, STDs, infectious diseases, tubal factor infertility, chronic inflammatory diseases, endometriosis, chronic endometritis, and endocrine disorders such as thyroid dysfunction, hypothalamic-pituitary disorders, previous reproductive tract surgeries, anemia, thrombophilia, current cancer or a medical history of familiar cancer and abnormal semen	Protocol: a RegenACR®-C Kit (Regen Laboratory, Le Mont-sur-Lausanne, Switzerland) PRP was prepared earlier on the day of administration. 60 mL of the patient’s peripheral blood was required in order to yield the required volume of PRP Platelet count 1,000,000 platelets/ µL Intramedullary injected on multiple sites in both ovaries with the patient under inhaled minimal sedation. The technique included penetration across the central part of each ovary respectively, gradual infusion of 4 mL of activated PRP, via a 17-gauge single lumen needle attached to the transvaginal probe transducer	Timing: random in amenorrheic POI and menopausal and Day 3 of cycle in POR and perimenopausal women. Immediately in women not receiving HR and stop HR for 6 months for women receiving HR Follow up for 3 months was done	Before and after assessment	Ovarian reserve markers Spontaneous pregnancy	No	None
Sills 2020 [30]	Single center USA	Quasi-experimental	182 POR	Inclusion criteria: had at least one ovary, infertility of > 1yr duration, at least one prior failed (or canceled) IVF cycle, or amenorrhea for at least three months Exclusion criteria: ongoing pregnancy, current or previous IgA deficiency, chromosomal ovarian insufficiency, prior major lower abdominal surgery resulting in pelvic adhesions, anticoagulant use for which plasma infusion is contraindicated, psychiatric disorder ongoing malignancy, or chronic pelvic pain	Volume: 8–10 mL whole blood was collected by peripheral venipuncture Centrifugation: 1500*g* × 5 min Processed blood was then fractionated, and erythrocytes were trapped beneath while lower density components settled atop the separator gel. Less than 3 mL of supernatant (corresponding to relatively platelet-poor plasma fraction) was then aspirated off the top of each column before recapping the vial for gentle tube inversion/resuspension PRP activation was achieved with calcium gluconate	10cc syringes were used to divide activated PRP samples into two equal portions and maintained at room temperature, then attached to a 35cm single lumen 19G needle assembly (Rocket Medical; Washington, UK). The injection apparatus was modified for office PRP administration by bypassing the Falcon tube collection port to allow direct injection into ovarian stroma under transvaginal ultrasound guidance. The ovaries were aligned with the needle guide to avoid intervening vascular or other structures and the needle was quickly advanced without rotation deep into the central ovary. Once tip placement was confirmed, activated substrate was slowly introduced as the needle was withdrawn across the previously traversed ovarian cortex. The final ~ 1mL of sample was deposited just under the ovarian capsule	Before and after assessment Follow up for 3 months was done	Ovarian reserve markers	NCT03178695	None
Stojkovska 2019 [31]	Single center Macedonia	Pilot comparative study	40 POR (ESHRE criteria) 20 PRP 20 control	Inclusion criteria: Age 53–42 years Normal semen analysis IVF completed with ET Exclusion criteria: Genetic or chromosomal ovarian insufficiency, immunoglobulin A deficiency, large surgical repairs of pelvic floor with severe pelvic adhesions, the use of anticoagulants, psychotropic medicaments, psychiatric disorders, carcinomas or a history of chronic pelvic pain, present infection, haemoglobin < 11 g/L or platelets < 150 × 10^3^/μL	Protocol: Regen PRP, (Regen Laboratory, Mont-sur-Lausanne, Switzerland) Under strict aseptic conditions and optimum temperature regulations (21–24°C), PRP was prepared according to the manufacturer’s guidelines	The volume immediately above the erythrocyte layer was collected. Calcium gluconate was used as an activator. After activation, in a period less than 2 min, approximately 3–5 ml of the PRP was injected into the ovaries under transvaginal ultrasound guidance 30 cm single lumen 17G aspiration needles under propofol intravenous anaesthesia	20 POR no intervention	FR IR CPR LBR	No	None
Tandulwadkar 2020 [32]	Single center India	Quasi-experimental	20 POR POSEIDON Group 3 and 4 (AFC < 5 and AMH < 1.1 ng/ml)	**Inclusion criteria:** Age 20–45 years Normal karyotype Normal semen parameters **Exclusion criteria:** Autoimmune diseases POI due to chemotherapy or radiotherapy Active viral infections	20 ml of peripheral blood in the heparinized syringe was taken and 2 ml of PRP was prepared after double centrifugation. This was mixed with 16 ml of ABMDSCs	Intraovarian instillation under general anesthesia of 6 ml (in younger patients with good volume of ovaries) or 4 ml (in women with inadequate ovarian volume) of ABMDSC’s per ovary at multiple sites along the long axis of the ovary starting from caudal end and continued by withdrawing the specially designed needle up to the cranial end into the main stroma. Injection was done under ultrasonographic guidance in 8 women and laparoscopically in 12 women	Before and after assessment All patients were followed up weekly for 6 weeks then underwent COS using minilong agonist protocol,	Ovarian reserve markers	No	None
Tulik 2022 [33]	Single center Turkey	Retrospective	71 women 50 POR (Bologna criteria 2 or more of age > 40 years; poor ovarian response in previous IVF cycles (≤ 3 oocytes retrieved; and abnormal ovarian reserve tests 21 POI ESHRE criteria at least 4 months of amenorrhea, FSH > 25 U/L and age < 40 years	Inclusion criteria: BMI 18–30 kg/m2 Exclusion criteria: endocrine disorders (thyroid dysfunction, hyperprolactinemia, diabetes mellitus, Addison disease, congenital adrenal hyperplasia, Cushing syndrome); corrected or present uterine anomalies; and azoospermia	Protocol: T-Biotechnology, Bursa, Turkey 20 mL of blood is collected from each patient into two tubes. Tubes are centrifuged at 1500 g for eight minutes. Approximately 2 mL of plasma is gathered above the newly formed buffy coat layer from each tube through a 16 G needle into a 5 mL syringe. Plasma obtained from the tubes is transferred into a single re-suspension tube and gently agitated for 30–60 s to prepare the PRP solution for use	A total of 4 mL of PRP solution was obtained per patient and divided into two equal portions to inject into each ovary. Patients were sedated for ovarian injection. The procedure was carried on with a 35 cm long 17 G needle under transvaginal ultrasound guidance. 2 mL of solution was injected into the stromal region of each ovary within two hours of PRP preparation	Before and after assessment AFC, menstrual pattern, and serum hormones were assessed monthly for at least 6 months	Cycle performance indicators (FR,IR,CPR,LBR, cancellation rate, no oocytes)	No	None

*CPR* Clinical pregnancy rate, *ChPR* Chemical pregnancy rate, *LBR* Live birth rate

Fourteen studies (1632 participants) were included in our analysis, 10 studies included women with POR [21, 23–28, 30–32], 1 study included women with POI [22] and 3 studies included both POR and POI women [20, 29, 33]. Among the included studies, 9 were Quasi-experimental [20–23, 26, 28–30, 32], 3 were retrospective [24, 27, 33] and 2 were case control studies [25, 31].

All the studies were conducted at a single center except Navali et al. [26]; Pacu et al. [27] that were conducted in 2 centers. Three studies were conducted in Iran [20, 24, 26], 3 in Turkey [22, 23, 33], 2 in USA [21, 30] and 1 study was conducted in each of the following countries, Greece [29], India [32], Macedonia [31], Romania [27], Ukraine [28] and Venezuela [25].

PRP volume injected was 0.2 ml in one study [25], 1 ml in 1 study [30], 2—4 ml in 9 studies, and 4–8 ml in 4 studies [21–23, 32]. The timing of PRP injection was random in all amenorrheic women and those with POI. In women without amenorrhea, PRP injection was done in day 1 -10 in 2 studies [22, 23], day 3–5 in 3 studies [21, 27, 29], day 7, 8 or 9 in one study [25], day 10 in one study [20], at time of follicular rupture in 1 study [24], at time of ovum pickup in 1 study [26] and not determined in 4 studies [28, 30–32]. The route in all studies was ultrasound guided transvaginal injection except in those with non-accessible ovarian who underwent laparoscopic injection.

### Risk of bias of included studies

Newcastle–Ottawa Scale was used to evaluate quality of the included studies (Table 2) and GRADE quality of evidence was separately done for each individual outcome criteria (Table 3).

**Table 2:** Quality assessment of the included studies using Newcastle–Ottawa Scale

[Study]	Selection	Comparability	Outcome /Exposure
Aflatoonian 2021 [20]	***	*	***
Barad 2022 [21]	***	*	***
Cakiroglu 2020 [22]	***	*	***
Cakiroglu 2022 [23]	***	*	***
Farimani 2021 [24]	***	*	**
Melo 2020 [25]	***	*	***
Navali 2022 [26]	***	*	**
Pacu 2021 [27]	**	*	**
Petryk 2020 [28]	***	*	*
Sfakianoudis 2020 [29]	***	*	***
Sills 2020 [30]	***	*	*
Stojkovska 2019 [31]	***	*	***
Tandulwadkar 2020 [32]	***	*	**
Tulik 2022 [33]	***	*	***

**Table 3:** GRADE quality of evidence

Outcome	No studies	Risk of bias	Inconsistency	Indirectness	Imprecision		Publication bias	Quality
					Sample size	Wide CI		
AMH	11	S	S	N	N	N	N	Low
FSH	9	S	S	N	N	N	N	Low
E2	4	S	S	N	S	S	N	Very Low
AFC	6	S	N	N	N	N	N	Moderate
Spontaneous pregnancy	5	S	N	N	S	S	N	Very Low
Number of oocytes retrieved	7	S	N	N	N	N	N	Moderate
Number of cleavage embryos	4	S	N	N	N	N	N	Moderate
Cancellation rate	3	S	N	N	S	N	N	Low
Fertilization rate	3	S	S	N	S	S	N	Very low
Clinical pregnancy rate	9	S	N	N	N	N	N	Moderate
Chemical pregnancy rate	3	S	N	N	S	N	N	Low
Live birth rate	7	S	N	N	N	N	N	Moderate

*AMH* Anti-Mullerian hormone, *AFC* Antral follicular count, *CI* Confidence Interval, *E2* Estradiol, *FSH* Follicle stimulating hormone, *N* Not serious, *S* Serious

### Synthesis of results

**Fig. 2:**
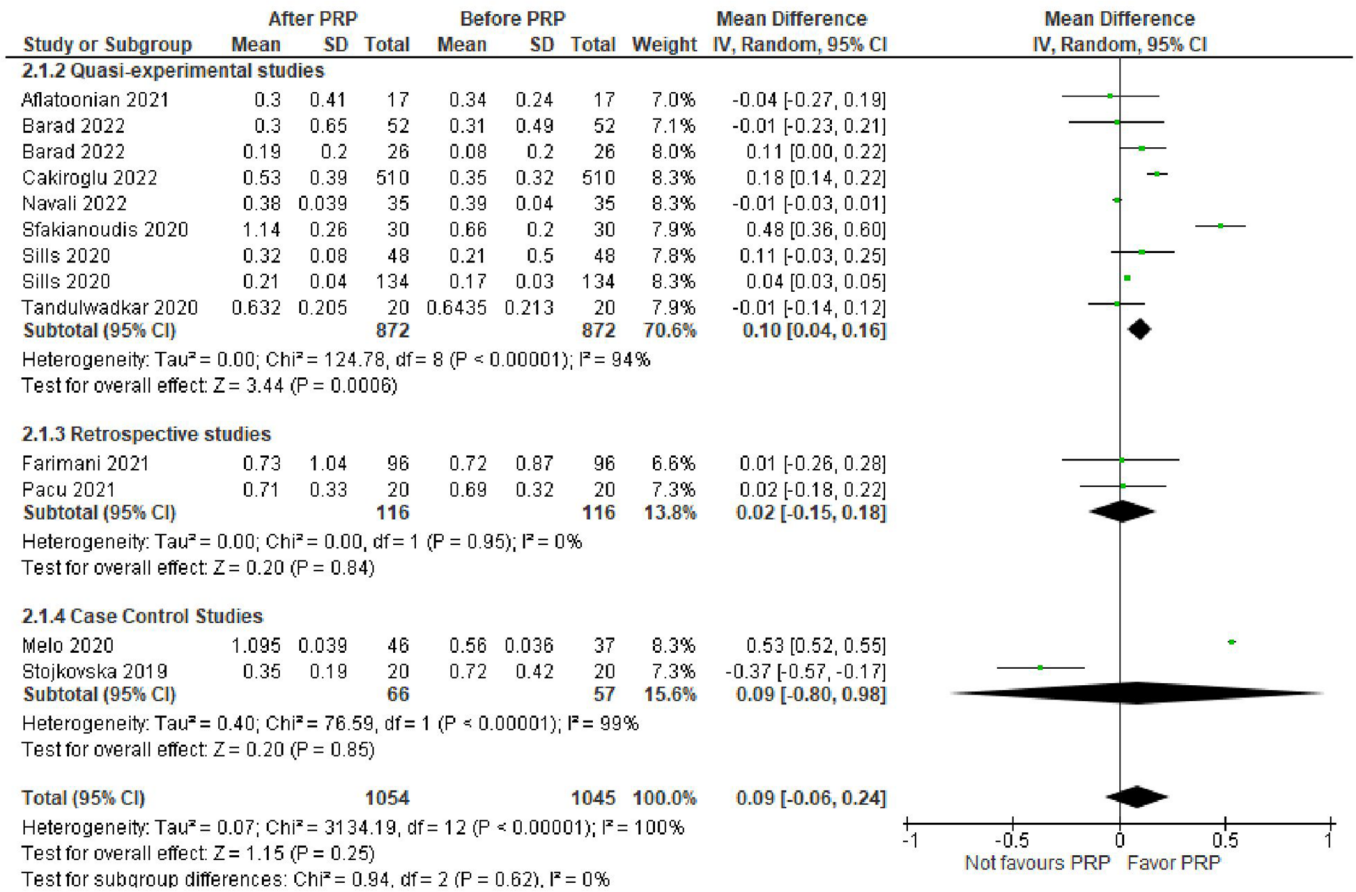
AMH level in included studies

Anti-Mullerian hormone (AMH) level was evaluated in 11 studies with 2099 POR women. The mean difference (MD) was 0.09 with 95% CI of – 0.06, 0.24 (*P* = 0.25). Subgroup analysis according to type of the involved studies revealed that AMH was reported in 7 Quasi-experimental studies (1744 women) with MD of 0.10 and 95% CI of [0.04, 0.16] (*P* < 0.001), 2 retrospective studies (232 women) with MD of 0.02 and 95% CI of [-0.15, 0.18] (*P* = 0.84) and 2 case control studies (123 women) with MD of 0.09 and 95% CI of [-0.80, 0.98] (*P* = 0.85) (Fig. 2).

**Fig. 3:**
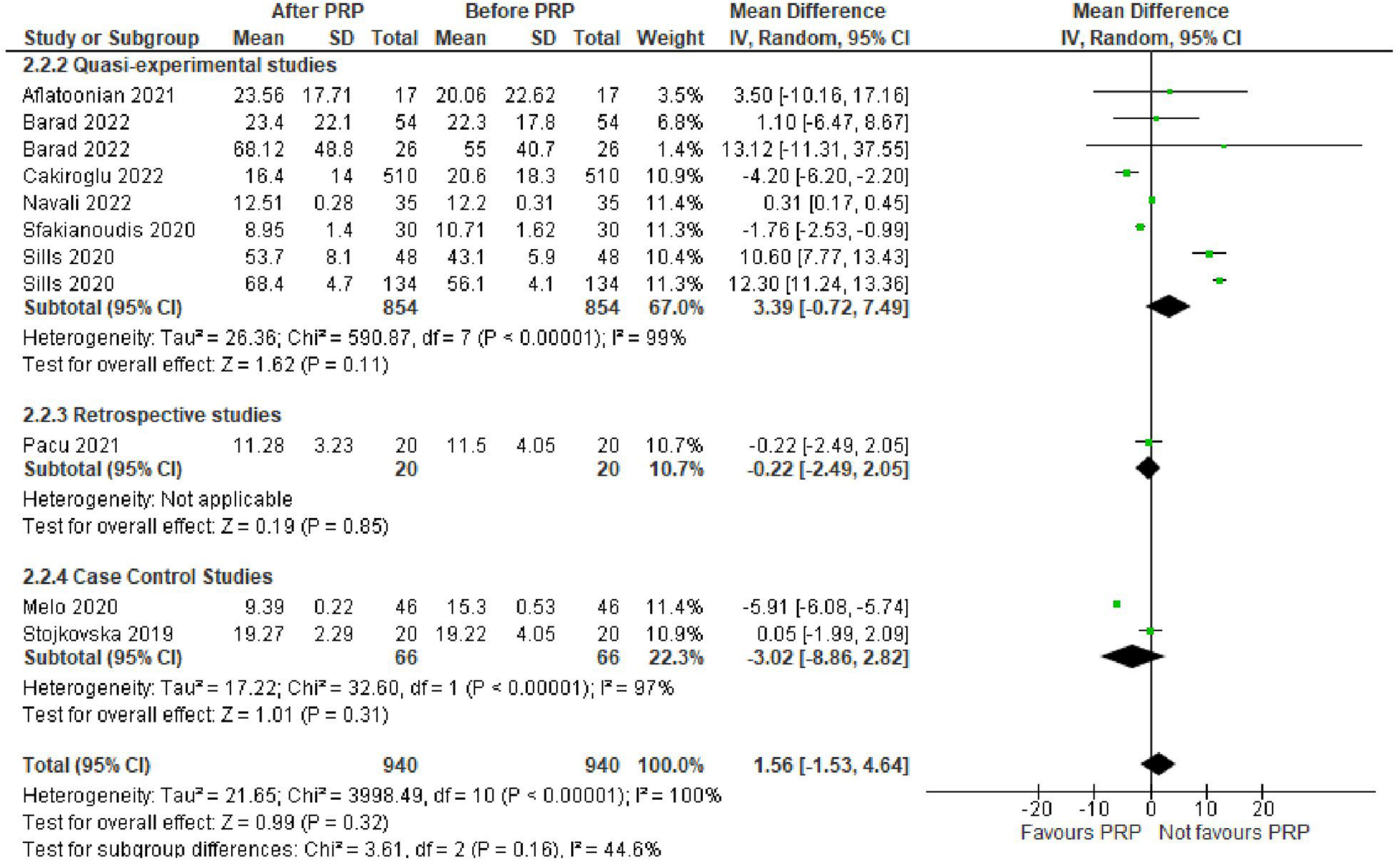
Basal FSH level in included studies

Basal Follicle stimulating hormone (FSH) level was assessed in 9 studies with 1880 POR women. The mean difference (MD) was 1.56 with 95% CI of – 1.53, 4.64 (*P* = 0.32). Subgroup analysis according to type of the involved studies revealed that FSH was reported in 6 Quasi-experimental studies (1708 women) with MD of 3.39 and 95% CI of [-0.72, 7.49] (*P* = 0.11), 1 retrospective study (40 women) with MD of -0.22 and 95% CI of [-2.49, 2.05] (*P* = 0.85) and 2 case control studies (132 women) with MD of -3.02 and 95% CI of [-8.86, 2.82] (*P* = 0.31) (Fig. 3).

**Fig. 4:**
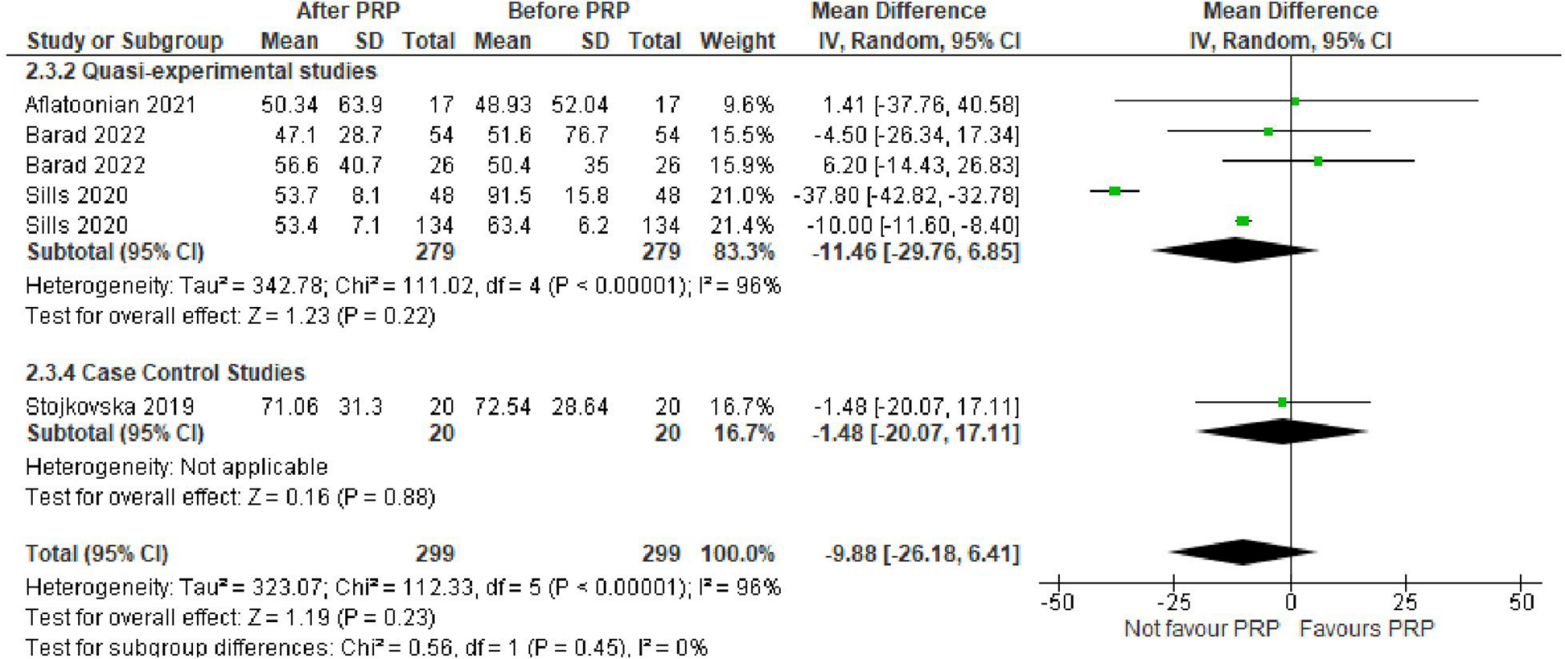
Basal E2 level in included studies

Basal serum estradiol (E2) level was assessed in 4 studies with 598 POR women. The mean difference (MD) was -9.88 with 95% CI of – 26.18, 6.41(*P* = 0.23). Subgroup analysis according to type of the involved studies revealed that basal E2 was reported in 3 Quasi-experimental studies (558 women) with MD of -11.46 and 95% CI of [-29.76, 6.85] (*P* = 0.22), and 1 case control study (40 women) with MD of -1.48 and 95% CI of [-20.07, 17.11] (*P* = 0.88) (Fig. 4).

**Fig. 5:**
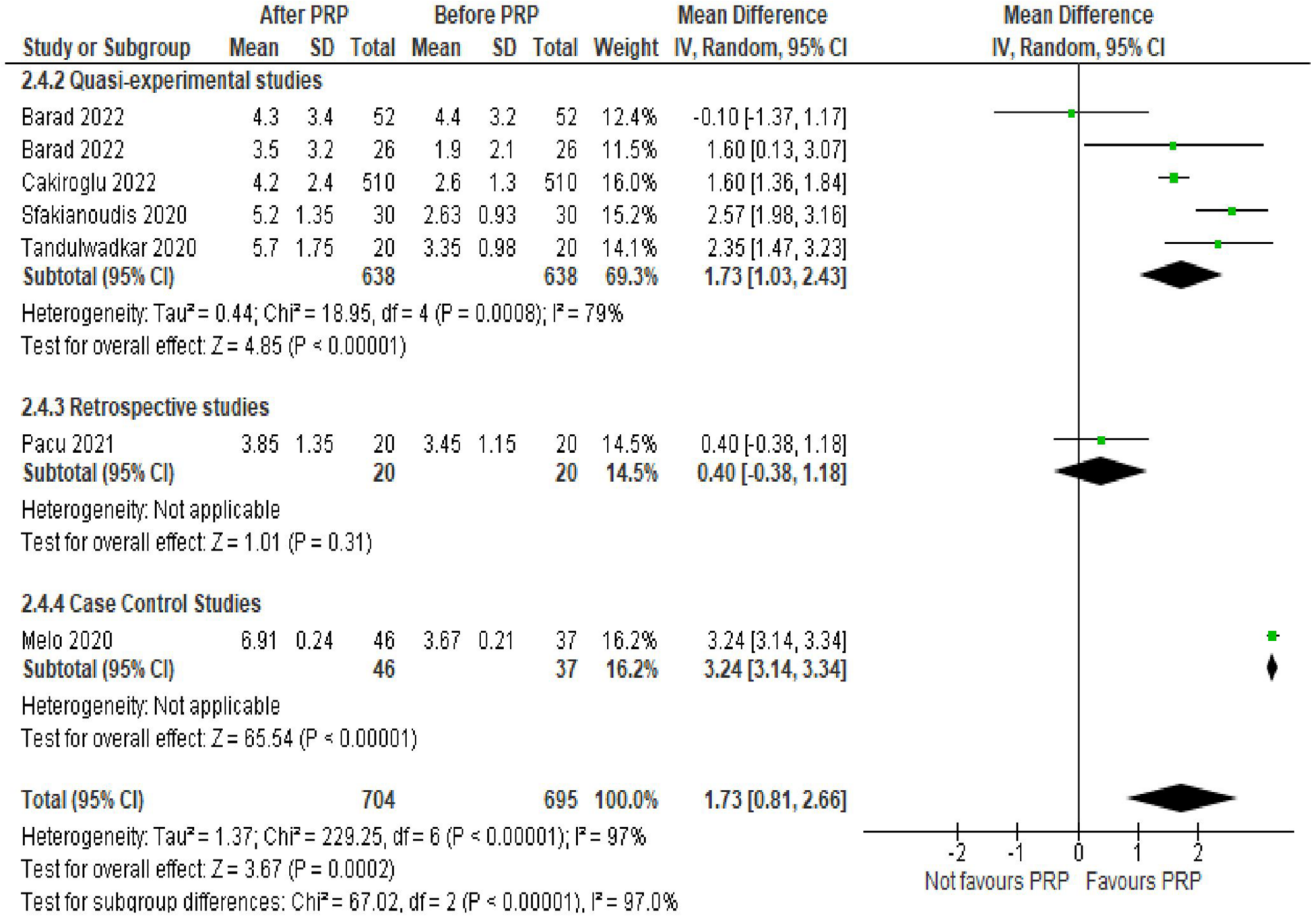
Antral follicular count in the included studies

Antral follicular count (AFC) level was assessed in 6 studies with 1399 POR women. The mean difference (MD) was 1.73 with 95% CI of 0.81, 2.66 (*P* < 0.001). Subgroup analysis according to type of the involved studies revealed that AFC was reported in 4 Quasi-experimental studies (1276 women) with MD of 1.73 and 95% CI of [1.03, 2.43] (*P* < 0.001), 1 retrospective study (40 women) with MD of 0.40 and 95% CI of [-0.38, 1.18] (*P* = 0.31) and 1 case control study (83 women) with MD of 3.24 and 95% CI of [3.14, 3.34] (*P* < 0.001) (Fig. 5).

**Fig. 6:**
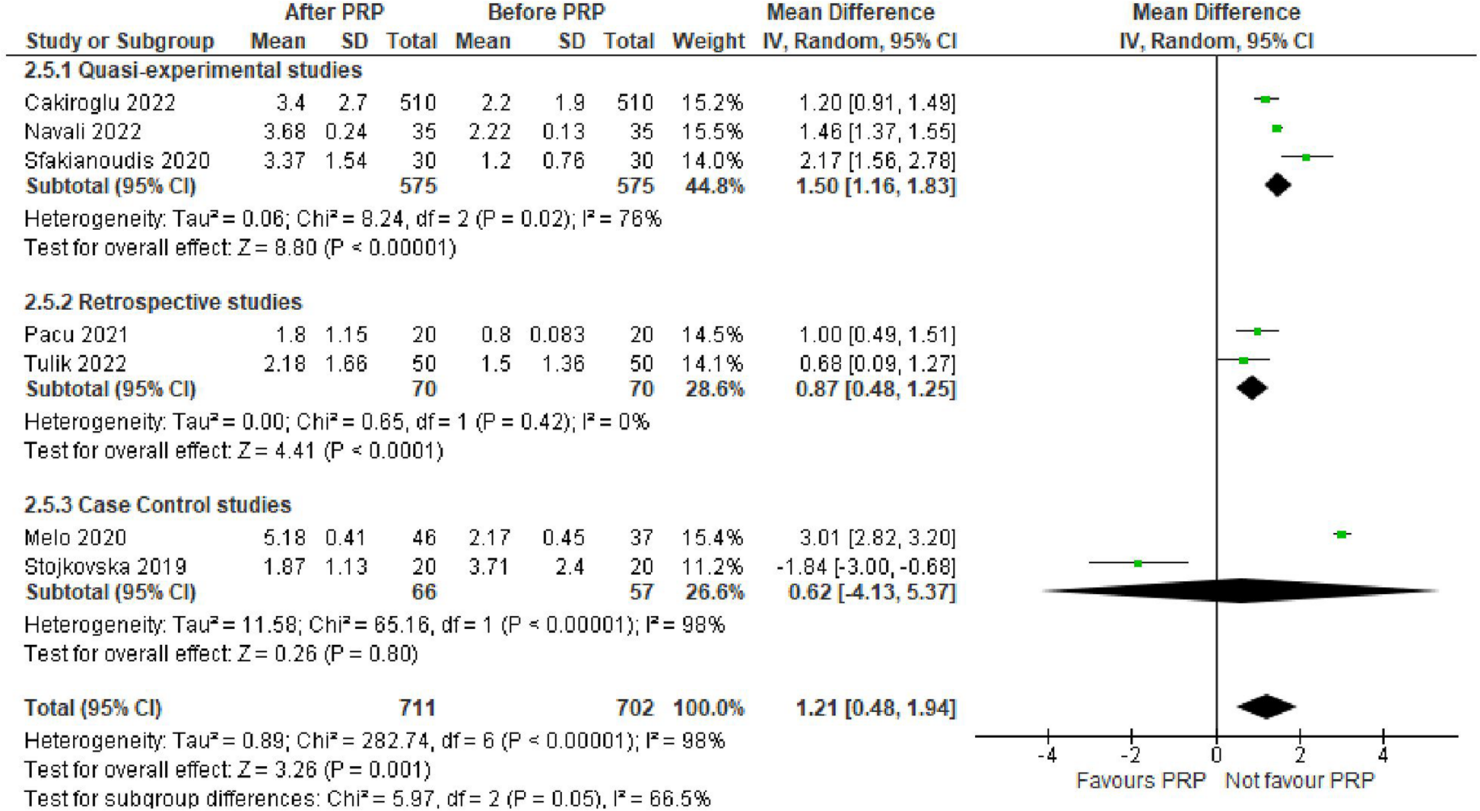
Number of oocytes retrieved in included studies

The number of oocytes retrieved was evaluated in 7 studies with 1413 POR women. The mean difference (MD) was 1.21 with 95% CI of 0.48, 1.94 (*P* = 0.001). Subgroup analysis according to type of the involved studies revealed that the number of oocytes retrieved was reported in 3 Quasi-experimental studies (1150 women) with MD of 1.50 and 95% CI of [1.16, 1.83] (*P* < 0.001), 2 retrospective studies (140 women) with MD of 0.87 and 95% CI of [0.48, 1.25] (*P* < 0.001)and 2 case control studies (123 women) with MD of 0.62 and 95% CI of [-4.13, 5.37] (*P* = 0.8) (Fig. 6).

**Fig. 7:**
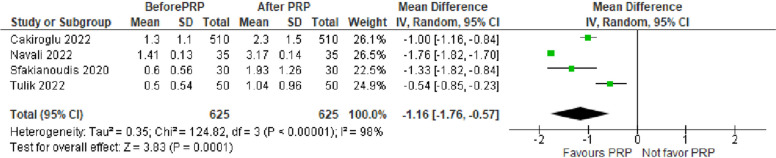
Number of cleavage embryos in included studies

The number of cleavage embryos was evaluated in 4 studies with 625 POR women. The mean difference (MD) was -1.16 with 95% CI of -1.76, -0.57 (*P* < 0.001) (Fig. 7).

**Fig. 8:**
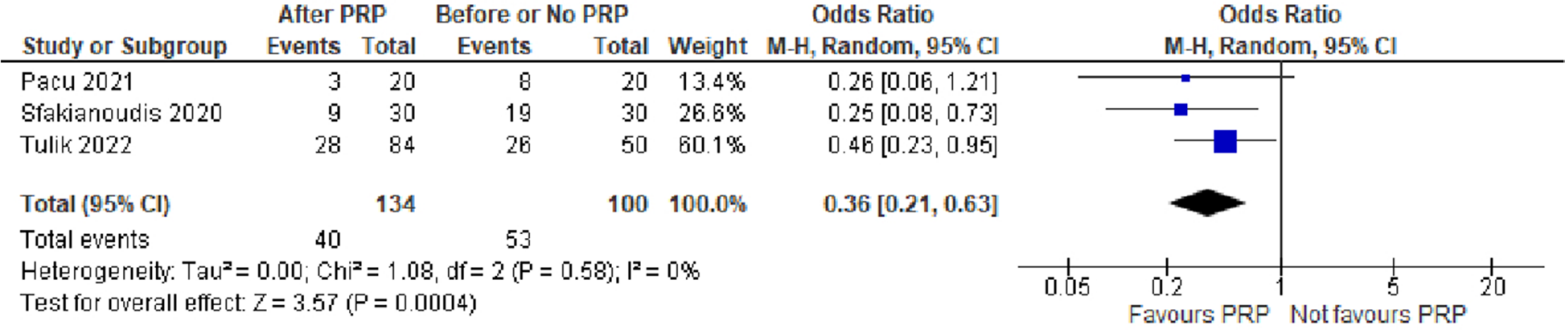
Cancellation rate in included studies

The cancellation rate was evaluated in 3 studies with 234 POR women. The Odds Ratio (OR) was 0.36 with 95% CI of 0.21, 0.63 (*P* < 0.001) (Fig. 8).

In women with POI, AMH and basal FSH levels were reported in 2 studies with 78 women and revealed a MD of 0.23 and -1.76 with a 95%CI of -0.29, 0.75 and-2.53, -1.0 and *P* values of 0.39 and < 0.001 respectively.

The rate of spontaneous pregnancy in both women with POR and POI are reported in Table 4.

**Table 4:** Outcome parameters reported among the included studies

Outcome	Type of participants	Study	Results
Number of good quality embryos	POR	Barad 2022 [21]	Before PRP 32.1% GI 14.9% GII After PRP 35.8% GI 7.4% GII
		Sfakianoudis 2020 [29]	Before PRP 8/18 (44.4%) After PRP28/58 (48.2%)
		Pacu 2021 [27]	Before PRP 0.33 ± 0.49 After PRP 0.76 ± 0.75 *P* value 0.073
Spontaneous pregnancy	POR	Aflatoonian 2021 [20]	After PRP 8/17
		Cakiroglu 2022 [23]	After PRP 22/510
		Navali 2022 [26]	After PRP 3/30
		Petryk 2020 [28]	After PRP 4/38
	POI	Aflatoonian 2021 [20]	After PRP 0/9
		Cakiroglu 2020 [22]	After PRP 23/311
		Sfakianoudis 2020 [29]	After PRP 8/55 (3/18 POI,4/24 perimenopausal, 1/13 menopausal)
Fertilization rate	POR	Cakiroglu 2022 [23]	Before PRP 57.6% After PRP 66.9% *P* value 0.008
		Tulik 2022 [33]	Before PRP 24/58 After PRP 104/144 *P* value 0.976
		Stojkovska 2019 [31]	Cases 80.67 ± 25.42 Control 65.6 ± 25.35 *P* value 0.44
	POI	Cakiroglu 2022 [23]	After PRP 55.8 ± 29.1
		Tulik 2022 [33]	After PRP 0.77 ± 0.72
Clinical pregnancy rate	POR	Cakiroglu 2022 [23]	After PRP 83/276
		Petryk 2020 [28]	After PRP 7/13
		Sfakianoudis 2020 [29]	After PRP 14/30
		Farimani 2021 [24]	After PRP 28/287
		Pacu 2021 [27]	After PRP 2/20
		Tulik 2022 [33]	Before PRP 1/50 After PRP 7/84 *P* value 0.16
		Melo 2020 [25]	Cases 11/46 Control 1/37
		Stojkovska 2019 [31]	Cases 33.33 ± 44.99 Control 10.71 ± 28.95 *P* value 0.69
	POI	Cakiroglu 2020 [22]	13/57 (7/28 fresh, 6/29 frozen ET)
Chemical pregnancy rate	POR	Barad 2022 [21]	After PRP 6/80
		Petryk 2020 [28]	After PRP 7/13
		Melo 2020 [25]	Cases 12/46 Control 2/37
Live birth rate	POR	Aflatoonian 2021 [20]	After PRP 4/17
		Barad 2022 [21]	After PRP 2/80
		Cakiroglu 2022 [23]	After PRP 12/510 spontaneous 54/276 IVF
		Petryk 2020 [28]	After PRP 6/13
		Sfakianoudis 2020 [29]	After PRP 12/30
		Pacu 2021 [27]	After PRP 2/20
		Tulik 2022 [33]	Before PRP 0/50 After PRP 4/84 *P* value 0.296
		Melo 2020 [25]	Cases 4/46 Control 1/37
		Stojkovska 2019 [31]	Before PRP 40 ± 50.71 14.29 ± 36.31 *P* value 0.71
	POI	Cakiroglu 2020 [22]	After PRP 16/311 spontaneous 9/87 IVF

*POI* Premature ovarian insufficiency, *POR* Poor ovarian response, *PRP* Platelet rich plasma

Table 4 summarized the number of good quality embryos, fertilization rate, clinical pregnancy rate, chemical pregnancy rate, and live birth rate in women with POR and those with POI. No meta-analysis was done for these outcomes as a result of marked heterogeneity and incomplete reporting (data were not completed after several emails to authors).

## Discussion

### Main findings

In our meta-analysis, we included 14 studies that evaluated the value of intraovarian injection of PRP in women with POR and POI.

Although there was an improvement of baseline hormones (AMH, FSH and E2) after intraovarian injection of PRP, this improvement failed to reach statistical significance (except the improvement of serum AMH analyzed in quasi-experimental studies).

This meta-analysis found a beneficial effect of intraovarian PRP injection on AFC (in quasi-experimental and case control studies), the number of retrieved oocytes (in quasi-experimental and retrospective studies), the number of cleavage embryos and the cancelation rate. These effects had moderate evidence regarding AFC, the number of oocyte retrieved and the number of cleavage embryos and low evidence regarding cancelation rate.

The effects of intraovarian PRP injection on clinical, chemical and live birth rate cannot be properly assessed as most of the included studies reported no data about these outcomes before PRP injection. However, the occurrence of spontaneous pregnancy, clinical pregnancy and live birth in women with POI reflects a significant change in these women.

The effects of PRP are linked to its high regenerative and anti-inflammatory properties. PRP was found to reduce inflammation, postoperative bleeding and infection. It also accelerates wound healing, osteogenesis and soft tissue healing [34].

The effect of PRP on AFC and the number of retrieved oocyte is more obvious than its effect on hormonal assessment. This may be explained by the physical recovery of the ovarian tissue that may precede its functional and hormonal recovery. Longer follow up may detect a functional recovery with improvement of the ovarian reserve hormone markers.

These tissue regenerative effects are linked to the growth factors contained in platelet granules. These growth factors include insulin-like growth factors, transforming growth factor-β, epidermal growth factor, and vascular endothelial growth factor [35].

These growth factors play important roles in cell migration, differentiation, and proliferation besides the activation of angiogenesis [36].

The inverse correlation between the concentration of growth hormone and growth factors with aging is documented in a previous study [37].

In a recent meta-analysis by Maged and colleagues in 2023, intrauterine and subendometrial injection of PRP were proved to improve the IVF cycle outcomes as implantation, clinical pregnancy, live birth rates and endometrial thickness in infertile women with previous implantation failure and those with refractory thin endometrium [19].

In rats with bilateral adnexal torsion, PRP injection was successful in prevention of ischemia and promotion of reperfusion through increase in growth factors, mainly VEGF [38].

### Strengths and limitations

This meta-analysis is the first comprehensive one evaluating the effects of intraovarian PRP injection in women with POR and POI. Although intraovarian PRP injection is a recent procedure, this meta-analysis included 14 studies. These represent all the available trials reached by extensive independent searching of all available published and unpublished. A separate analysis was done for POR and another one for women with POI. Adequate subgroup analysis according to different study designs for all the available outcomes was done.

This meta-analysis is not without limitations. None of the included studies was RCT, so it carries a high risk of bias. Most of the studies did not report the clinically significant outcomes such as clinical pregnancy and live birth rates. Even the studies that reported these outcomes failed to compare them either to before intervention nor to controls. There is marked heterogeneity among the included studies regarding the study design, baseline hormonal levels, timing of PRP injection, the time for the outcomes assessment and reporting of outcomes. We used the random effect method for comparison to compensate for this heterogeneity. The data may be limited by the fact that some of the patients included have received concomitant other treatments.

Despite these limitations that were expected as this line of treatment is recently introduced in the field of infertility, the promising findings of our study encourage the conduction of a well-designed randomized control study with proper selection criteria and low risk of bias to confirm these results.

### Comparison with existing literature

Although there are many systematic reviews conducted to assess the benefits of PRP in skin, eye and bone diseases, only a few studies were conducted on infertility. Only one systematic review studied intraovarian PRP in women with POR or ovarian Insufficiency [39].

However, this systematic review included only 4 studies. Most of them did not evaluate pregnancy characteristics as clinical pregnancy, chemical pregnancy, or live birth rates. They failed to conduct a meta-analysis of 4 studies with marked heterogeneity. Also, this review lacks any subgroup analysis.

Our meta-analysis suggests that intraovarian PRP injection could be tried in all women with POI and those with POR in whom other measurements to improve their ovarian response failed. PRP is relatively a safe procedure that improves the ovarian response and function. With progress in preparation of PRP and addition of other stimulatory, growth factors and stem cells, it can provide future hope for fertility in those women suffering from POI.

## Conclusions

This systematic review found a non-significant improvement in ovarian hormones (AMH, basal FSH or basal E2) and a significant improvement of AFC, the number of retrieved oocytes, the number of cleavage embryos and the cancellation rate. However the quality of evidence of these findings was not high. A well designed RCT with adequate blinding, with properly selected inclusion criteria considering the level of ovarian reserve markers should be conducted to provide the needed evidence. Also setting an optimum level of different ovarian reserve markers to achieve the maximum benefits from intraovarian PRP injection is recommended.

### Supplementary Information

**Additional file 1: Supplementary Table S1.** Search strategy.

## Data Availability

Data used and/or analised during the study are available from the corresponding author upon reasonable request.
