# Supplementary material for: The value of intraovarian autologous platelet rich plasma in women with poor ovarian reserve or ovarian insufficiency: a systematic review and meta-analysis
Source: BMC Pregnancy Childbirth. 2024 Jan 27;24:85. doi: 10.1186/s12884-024-06251-2 (PMC10821562; doi:10.1186/s12884-024-06251-2)
Supplement: Supplementary file 1 — Additional file 1: Supplementary Table S1. Search strategy. [file 12884_2024_6251_MOESM1_ESM.docx]

Supplementary table S1 Search strategy

| Database | Keywords | Results |
| --- | --- | --- |
| Medline | (Premature ovarian failure [MeSH]OR Decreased ovarian reserve [MeSH]) AND (platelet-rich plasma OR PRP OR Autologous platelet-rich plasma) | 505 |
| Embase, | ((Premature ovarian failure OR Decreased ovarian reserve OR premature menopause OR Poor ovarian reserve) AND (platelet-rich plasma OR PRP OR Autologous platelet-rich plasma) | 113 |
| Web of Science | ((Premature ovarian failure OR Decreased ovarian reserve OR premature menopause OR Poor ovarian reserve) AND (platelet-rich plasma OR PRP OR Autologous platelet-rich plasma) | 84 |
| Scopus | Ovarian reserve AND platelet rich plasma | 624 |
| the Cochrane Central Register of Controlled Trials | (Ovarian reserve OR ovarian failure) AND ((platelet-rich plasma OR PRP) | 559 |
